# Supplementary material for: Coaching doctors to improve ethical decision-making in adult hospitalized patients potentially receiving excessive treatment: Process evaluation study of the CODE intervention in doctors and nurses working in ten acute hospital wards
Source: PLoS One. 2025 Dec 8;20(12):e0337801. doi: 10.1371/journal.pone.0337801 (PMC12685199; doi:10.1371/journal.pone.0337801)
Supplement: S1 Table — (DOCX) [file pone.0337801.s001.docx]

Online Supplement

**S1 Table. Characteristics of participants of the interview studies**

| Focus group | Wave | Discipline | Gender | Age |
| --- | --- | --- | --- | --- |
| FG1 | 1 | Junior doctor | male | 30-39 |
| FG1 | 1 | Junior doctor | female | 20-29 |
| FG2 | 1 | Deputy head Nurse | female | 40-49 |
| FG2 | 1 | Nurse | female | 20-29 |
| FG2 | 1 | Nurse | female | 30-39 |
| FG2 | 1 | Nurse | female | >50 |
| FG3 | 1 | Senior doctor | male | >50 |
| FG3 | 1 | Senior doctor | female | >50 |
| FG3 | 1 | Senior doctor | male | >50 |
| FG4 | 1 | Senior doctor | female | 30-39 |
| FG4 | 1 | Senior doctor | female | 30-39 |
| FG4 | 1 | Senior doctor | male | 40-49 |
| FG5 | 1 | Nurse | female | >50 |
| FG5 | 1 | Nurse | female | 30-39 |
| FG5 | 1 | Nurse | female | 20-29 |
| FG5 | 1 | Nurse | female | 30-39 |
| FG5 | 1 | Nurse | female | 40-49 |
| FG5 | 1 | Nurse | female | 40-49 |
| FG5 | 1 | Nurse | female | 40-49 |
| FG6 | 2 | Nurse | female | 40-49 |
| FG6 | 2 | Nurse | female | 40-49 |
| FG6 | 2 | Nurse | female | 40-49 |
| FG6 | 2 | Nurse | female | 30-39 |
| FG6 | 2 | Nurse | female | 40-49 |
| IV1 | 2 | Nurse | female | 30-39 |
| FG7 | 2 | Head nurse | female | > 50 |
| FG7 | 2 | Deputy head nurse | female | 40-49 |
| FG7 | 2 | Head nurse | male | 40-49 |
| IV2 | 2 | Junior doctor | male | 30-39 |
| FG8 | 2 | Senior doctor | male | >50 |
| FG8 | 2 | Senior doctor | female | 40-49 |
| FG8 | 2 | Senior doctor | male | 30-39 |

*Note.* FG = Focus group; IV = Interview
