# Supplementary material for: Coaching doctors to improve ethical decision-making in adult hospitalized patients potentially receiving excessive treatment: Process evaluation study of the CODE intervention in doctors and nurses working in ten acute hospital wards
Source: PLoS One. 2025 Dec 8;20(12):e0337801. doi: 10.1371/journal.pone.0337801 (PMC12685199; doi:10.1371/journal.pone.0337801)
Supplement: S1 File — (DOCX) [file pone.0337801.s003.docx]

**Unofficial translation in English of the ethics approval document**

Concerning: Advise for the monocentric study titled ‘Coaching doctors in ethical decision-making : a stepped wedge cluster randomization study in 10 departments of the Ghent University Hospital (CODE-study)

B.U.N.: B6702021000439

*Covering letter dd 14/4/2021

*Advice request form document A Version 1.0 dd 8/03/2021

*Protocol CODE Study Version 1.0 dd 9/03/2021

*Patient information and consent form:

Focusgroup Clinician Version 1.0 dd 8/03/2021

Family Version 1.0 dd 8/03/2021

Surrogate decision-maker Version 1.0 dd 8/03/2021

Doctor Version 1.0 dd 8/03/2021

Head of department and head nurse Version 1.0 dd 8/03/2021

Patient Version 1.0 dd 8/03/2021

Nurse Version 1.0 dd 8/03/2021

*Questionnaires Version 1.0 dd 8/03/2021

*CV

Prof Dr Dominique Benoit

Prof Dr Ruth Piers

*GCP certificate

Prof Dr Ruth Piers dd 7/2/2020

Prof Dr Dominique Benoit dd 2/6/2020

Advice was asked by : Prof dr. Dominique Benoit

The above mentioned documents have been reviewed by the Ethics Committee.

A positive advice was given for this protocol on 25/05/2021. In case the study is not started by 25/05/2022, this advice will be no longer valid and the project must be resubmitted.

Before initiating the study, please contact HIRUZ CTU (093320500).

*The Ethics Committee is organized and operates according to the ‘ICH Good Clinical Practice’ rules.

*The Ethics Committee stresses that approval of the study does not mean that the Committee accepts responsibility for it. Moreover, please keep in mind that your opinion as an investigator is presented in the publications, reports to the government, etc., that are a result of this research.

*In the framework of ‘Good Clinical Practice’, the pharmaceutical company and the authorities have the right to inspect the original data. The investigators have to assure that the privacy of the subjects is respected.

*The Ethics Committee stresses that it is the responsibility of the promoter to guarantee the conformity of the non-dutch informed consents forms with the dutch documents.

*None of the investigators involved in this study is a member of the Ethics Committee.

*All effective members of the Ethics Committee or their representatives have reviewed this project. (The list of the members is enclosed).

**Signed on behalf of the Ethics Committee by**

**Prof. dr. P Deron**

**Chairman**

Membership List as of 25/05/2021

Chairperson: Prof. Dr. P. Deron
Secretary: Prof. Dr. R. Peleman

| **Full Member** | **Alternate Member** |
| --- | --- |
| Dr. G. VAN LANCKER (UZG – clinical pharmacologist, ♀) | Prof. Dr. S. ROTTEY (UZG – clinical pharmacologist, ♀) |
| Prof. Dr. D. DE BACQUER (UG – statistician, ♂) | Prof. Dr. P. COOREVITS (UG – statistician, ♂) |
| Dr. J. VAN ELSEN (general practitioner, ♂) | Dr. M. COSYNS (general practitioner, ♂) |
| Prof. Dr. K. DE GROOTE (UZG – pediatric cardiologist, ♀) | Prof. Dr. P. SCHELSTRAETE (UZG – pediatric pulmonologist/infectiologist, ♀) |
| Prof. Dr. W. NOTEBAERT (UG – psychologist, ♂) | Mr. W. SCHRAUWEN (UZG – psychologist, ♂) |
| Mrs. M. FOUQUET (UZG – nurse, ♀) | Mrs. I. VLERICK (UZG – nurse, ♀) |
| Mr. C. DEMEESTERE (UZG – nurse, licentiate in Medical Social Sciences, ♂) | Mr. G. DE SMET (UZG – nurse, licentiate in Medical Social Sciences, ♂) |
| Mrs. K. KINT (UZG – pharmacist, ♀) | Mrs. L. HUYS (UZG – pharmacist, ♀) |
| Mr. B. VANDERHAEGEN (UZG – moral theologian, ♂) | Prof. Dr. S. STERCKX (UG – moral philosopher, ♀) |
| Prof. Dr. Mr. T. BALTHAZAR (UG – legal expert, ♂) | Prof. Dr. T. GOFFIN (UG – legal expert, ♂) |
| Mrs. C. VANCAENEGHEM (patient representative, ♀) | Mrs. S. DE GROOTE (patient representative, ♀) |
| Prof. Dr. P. DERON (UZG – surgeon, ♂) | Prof. Dr. W. CEELEN (UZG – surgeon, ♂) |
| Prof. Dr. R. PELEMAN (UZG – internist/pulmonologist, ♂) | Prof. Dr. H. VERSTRAELEN (UZG – vulva specialist, ♂) |
| Prof. Dr. J. DECRUYENAERE (UZG – internist/intensivist, ♂) | Dr. N. PETERS (IJZG – fertility doctor, ♀) |
| Prof. Dr. R. RUBENS (UZG – internist/endocrinologist, ♂) | Prof. Dr. W. VAN BIESEN (UZG – nephrologist, ♂) |
| Prof. Dr. M. DE MUYNCK (UZG – physical medicine and rehabilitation specialist, ♀) | Dr. S. JANSSENS (UZG – geneticist, ♀) |
| Prof. Dr. K. DHONDT (UZG – (child) psychiatrist, ♀) | Dr. L. GOOSSENS (UZG – neonatologist, ♀) |

UZG = Ghent University Hospital, UG = Ghent University

Assessment is carried out by the full members. If a full member is unable to assess, the assessment is carried out by his/her alternate member.
